# Research Status and Future Development of Cochlear Reimplantation

**DOI:** 10.3389/fnins.2022.824389

**Published:** 2022-03-21

**Authors:** Xinyi Yao, Haotian Liu, Jinyuan Si, Xiuyong Ding, Yu Zhao, Yun Zheng

**Affiliations:** ^1^Department of Otolaryngology-Head and Neck Surgery, West China Hospital of Sichuan University, Chengdu, China; ^2^Department of Otolaryngology-Head and Neck Surgery, XuanWu Hospital of Capital Medical University, Beijing, China

**Keywords:** cochlear implants, reimplantation, literature review, revision surgery, reimplantation rate

## Abstract

Cochlear implants are the most successful sensory prostheses worldwide, and they can be useful for patients with severe and profound hearing impairment. However, various complications, including infection, pain, and device failure which is mainly due to falls and trauma, are associated with the use of cochlear implants. Reimplantation is required to replace the initial device in severe complications. Nevertheless, reimplantation can present certain surgical risks and may impose a significant economic and psychological burden on patients and their families; therefore, it requires greater attention and focus. This article presents a review of the literature on cochlear reimplantation and summarizes the current status, knowledge gaps, and future research directions on cochlear reimplantation. Since 1980s, cochlear reimplantation techniques can be considered to be relatively mature; however, some clinical and scientific problems remain unresolved, including the lack of a unified definition of cochlear reimplantation, non-standardized calculation of the reimplantation rat, and insufficient effect assessment. This review highlights the urgent need to establish an international consensus statement on cochlear reimplantation research to standardize the definition, calculation formulas of reimplantation rate, and follow-up systems.

## Introduction

Cochlear implants (CIs), the most successful sensory prostheses worldwide, are small but complex electronic devices implanted in the cochlea that can help patients with severe or profound hearing impairment hear external sound (Büchner and Gärtner, 2017). Since the 1970s, continuous advancements have been improved the speech-coding strategies, electrodes, and materials used for developing CIs (Büchner and Gärtner, 2017; Carlyon and Goehring, 2021). The indications for CIs have also expanded gradually, and the number of patients with CIs has been increasing (Lailach et al., 2021).

Cochlear reimplantation has also attracted increasing public attention because of the various complications associated with the use of CIs, including skin flap infection, electrodes migration, and device failure which are mainly caused by falls and trauma (Weise et al., 2005; Terry et al., 2015; Dağkıran et al., 2020). Cochlear reimplantation surgery is an invasive operation, and CIs are very expensive. Thus, reimplantation may greatly increase the economic and psychological burden on patients and families, especially in those living in developing countries where CIs are not covered by medical insurance (Sorkin and Buchman, 2016; Qiu et al., 2017; Chen et al., 2021).

Moreover, some patients showed poorer hearing and speech recognition levels after reimplantation than those before initial implantation (Chung et al., 2010; Balakina et al., 2015; Manrique-Huarte et al., 2015), indicating the need for greater attention to this population. Nevertheless, only a few studies have summarized the current status of research on cochlear reimplantation, and there are no studies or reviews on the future research directions of cochlear reimplantation. This review aims to summarize the history and current state of knowledge of cochlear reimplantation, enumerate the research gaps and suggest directions for future research.

## Part I: History and Current Status of Cochlear Reimplantation

In Hochmair-Desoyer and Burian (1985) first reported the use of cochlear reimplantation in two adults with post-lingual deafness in Austria (Desoyer and Burian, 1985). The effect of cochlear reimplantation was assessed using Bekesy threshold tracking, loudness scaling, and open lists of unknown single-syllable words and daily sentences, among other techniques. The patients showed no significant changes in hearing thresholds or speech recognition between pre- to post-reimplantation. In Burian and Eisenwort (1989) studied four prelingually deaf children who underwent reimplantation and found that speech discrimination levels remained the same or even improved after reimplantation. In the same year, Spillmann and Dillier (1989) reported a case of reimplantation for device failure, in which a single-channel CI was upgraded to a multichannel CI. The device upgrade resulted in a significant improvement in the auditory performance of the patient. These early studies provided preliminary evidence confirming the satisfactory effects of reimplantation.

Two CI surgeries were performed on the same ear of eight adult cats (Robert et al., 1989). They found that the initial electrodes could be removed easily and that new electrodes could be implanted successfully without damaging the peripheral cochlear nerve; however, the proliferation of granulation tissue in round windows and scala tympani may lead to difficulties in implanting new electrodes. In addition, Jackler et al. (1989) was the first to assess manufacturer-specific cochlear reimplantation rates, i.e., Cochlear Corp., 2.8%; Richards Corp., 3.3%; Storz Corp., 7%. In Webb et al. (1991) who collated cochlear implantation complication data from Germany, Australia, and the United States, reported the number of reimplanted CIs. In Maas et al. (1996) first proposed a new reimplantation strategy, in which the contralateral ear was chosen for reimplantation, and the postoperative performance on speech recognition tests remained the same or improved.

Since 2000, CI technology has been advanced by successive multicenter cohort studies. For example, Parisier et al. (2001) reviewed the operations and postoperative findings for initial implantation and reimplantation in 25 children and reported the postoperative complications of reimplantation, which demonstrated that reimplantation in children is feasible and effective. From 2005 to 2018, an increasing number of new and special cases of reimplantation have been reported, such as reimplantation for various flap infections, intractable facial nerve stimulation, and reimplantation in patients with inner ear malformations (Puri et al., 2005; Polak et al., 2006; Ahn et al., 2008; Incesulu et al., 2008). In addition, long-term large-scale cohort studies have been undertaken. In a study of 18 hospitals across the United States, Henson et al. tracked and collected the information on 22 patients who had undergone reimplantation and found that approximately 60% of the patients had better or similar auditory outcomes after reimplantation than after the initial implantation (Henson et al., 1999). Weder et al. (2020) conducted the longest study, to date, from 1982 to 2018, in which a tertiary referral hospital performed 4,600 initial cochlear implantations and 22 reimplantations due to infection. They found that speech recognition after reimplantation was comparable to that before reimplantation. However, the reimplantation rate reported by Weder’s study was only 0.48%, which does not represent the overall reimplantation situation, as patients who underwent reimplantation for device failure and other reasons were not included. The largest sample size was reported in a multicenter study conducted by Hermann et al. (2020) who described the findings for 4,952 initial cochlear implantations and 99 reimplantations, with a 2% reimplantation rate. Of the 99 cases of reimplantation, only one had postoperative infection after reimplantation.

The number and quality of CIs in China have been gradually increasing since the first adult multichannel cochlear implantation was performed at the Peking Union Medical College Hospital in 1995 (Han, 2004; Xi et al., 2005). In 2010, more than 10,000 CIs were implanted in China, leading the rest of the world in terms of the number of procedures performed (Cao and Wei, 2010). In 2018, the total number of children with CIs exceeded 50,000 (Lu, 2018). Meanwhile, the benefits of CIs to Chinese speech recognition of implanted patients have been widely reported (Luo et al., 2008; Chang et al., 2016). The reimplantation surgery in China was first reported in a study of six patients, with a reimplantation rate of 16.67% (Yu et al., 2004). In Zhao et al. (2008) evaluated the effects of reimplantation from the perspective of effective working electrodes. To date, more than 40 studies on reimplantation in China have been published in Chinese, and six have been published in English. These studies have primarily focused on cause analysis, surgical discovery, and the management of complications.

## Part II: Current Deficiencies and Future Research Directions

### Non-standard Definitions of Cochlear Reimplantation

Currently, the primary concern in cochlear reimplantation is the lack of an internationally unified and normative definition. In Jackler et al. (1989) defined cochlear reimplantation as the removal of an indwelling CI electrodes followed by reinsertion of a new device. They noted that reimplantation is a maneuver of uncertain consequences to the cochlea and its surviving nerve (Robert et al., 1989).

However, according to a literature review, most researchers have not clearly defined cochlear reimplantation (Orús et al., 2010; Ciorba et al., 2012). Some articles did not attempt to define cochlear reimplantation at all (Bhadania et al., 2018; Batuk et al., 2019). “Reimplantation/re-implantation” are often used to mean CI reimplantation, and “reinsertion/replacement” are also used in the same context (Desoyer and Burian, 1985; Parisier et al., 1991; Holcomb et al., 2018; Lane et al., 2019). However, there existed confusion and genericity between “revision” and “reimplantation” in many studies (Lassig et al., 2005; Rivas et al., 2008; Hwang et al., 2019). For example, Marlowe et al. proposed that revision surgery is a means to deal with abnormal implantation sites or internal problems with the implanted device (Marlowe et al., 2010). Revision surgery has been defined as the removal of the old device and replacement with a prosthesis, which is similar to the definition of cochlear reimplantation.

However, the procedures performed to treat CI complications were classified as follows: (1) cochlear reimplantation, (2) other revision surgery, and (3) medical treatment (Lescanne et al., 2011; Tarkan et al., 2013). Therefore, we speculate that cochlear reimplantation is characterized by replacing the initial electrodes with brand-new devices, and that revision surgery comprises surgical operations performed to address CI complications. Thus, cochlear reimplantation is a part of revision surgery.

Overall, the first requirement for future studies is to formulate a scientific and rigorous definition of reimplantation to standardize relevant studies and enhance comparability. In this regard, it is imperative to establish a global committee comprising cochlear implant manufacturers, FDA authorities, and clinicians/academicians from a variety of settings to propose an international consensus on cochlear reimplantation to standardize its definition.

### Unclear Range of Cochlear Reimplantation

The second important issue is the unclear range of cochlear reimplantation. In Jackler et al. (1989) were the first to systematically summarize the following reasons for reimplantation: device failure, flap infections, electrodes mis-insertion or compression, hematoma or trauma at the receiver site, and accidental displacement (Robert et al., 1989). The European Consensus Statement published in 2005 classified the reasons as device failure, medical reason, characteristic decrement, and performance decrement (No authors listed, 2005). Device failure can be divided into hard and soft failures based on whether the failure can be proven with *in vivo* tests (Balkany et al., 2005). This was the first unified document regarding cochlear reimplantation. However, the 2005 consensus did not clearly propose a definition and scope of cochlear reimplantation and lacked procedures and tools for screening and the classification of the reasons.

In Battmer et al. (2010) published the International Classification of Reliability for Implanted Cochlear Implant Receiver Stimulators, which adopted a similar framework as the 2005 European Consensus. This framework illustrated some details, such as the CI survival time, (reduced) clinical benefit, and specification (Battmer et al., 2010). In 2017, the Association for the Advancement of Medical Instrumentation (AAMI) updated and standardized the classification of explanted CIs (Zwolan and Verhof, 2017) and proposed the following four categories: medical reasons, non-medical reasons, inconclusive/no faults found, and combined reasons. Most studies have adopted these three classification categories (Hermann et al., 2020; Layfield et al., 2021). However, there is no unified research range for cochlear reimplantation.

According to a literature review, most studies regarded the following situations as reimplantation: (1) removal of the initial electrodes for various reasons and implantation of a new device on the ipsilateral or contralateral side, which was the most common scenario defined as reimplantation (Lassig et al., 2005); (2) replacement of the failed hybrid CI with short electroacoustic stimulation (EAS) electrodes or full-length electrodes (Kamat et al., 2011; Jayawardena et al., 2012; Dunn et al., 2015); (3) reinsertion of the initial electrodes into the cochlea in cases of device migration (Luo et al., 2020); (4) reinsertion of the initial electrodes into the cochlea on the operation day or within a few days of the operation due to the electrodes in incorrect insertion places, such as the internal auditory canal, eustachian tube, vestibul. (Gözen et al., 2019); and (5) simultaneous implantation on both sides when patients with unilateral CI accept reimplantation surgery (Tang et al., 2019).

However, these classifications are not entirely appropriate. We consider that the first kind falls in the range of cochlear reimplantation, which replaced the initial device with a brand new one. And the second one can be regarded as a sort of reimplantation as well, which is relatively rare. For the third and fourth definitions, no new devices were inserted into the cochlea; thus, they cannot be regarded as reimplantation. For the fifth definition, ipsilateral implantation with a new device can be considered as reimplantation; however, contralateral implantation was the first CI, not the reimplanted CI. In conclusion, future studies should propose a more precise scope and classification of cochlear reimplantation.

### Non-uniform Calculation of Cochlear Reimplantation Rate

The third important issue is the calculation formula of CI reimplantation rate, which varies greatly across studies. In Battmer et al. (2010) proposed the definition and calculation of the cumulative survival rate (CSR), which was in accordance with the methodology outlined in ISO standard 5841-2:2000 and targeted device failure without accounting for the medical reasons. In 2017, AAMI published the definition and calculation of the cumulative removal percentage (CRP), which covered all explanted CIs (Zwolan and Verhof, 2017). However, the formulation of CRP is not applicable to cochlear reimplantation rate. Cochlear removal mainly refers to the explantation of existing devices due to device failure, medical reasons, and inconclusive defects. In contrast, cochlear reimplantation is defined as the explantation of the initial device, followed by implantation of new electrodes. However, not all patients undergoing explantation of ordinary devices received new devices, and in some cases, the failed device was left *in situ*, while the new device was implanted on the contralateral side. These issues highlight the importance of standardizing the calculation formulation for the CI rate based on the CRP.

According to a literature review, some studies have used the number of patients as the unit of measurement, regardless of whether they had undergone unilateral and bilateral implantation (Sterkers et al., 2015), Dotú et al. (2010), whereas others have used the CI number as the unit of measurement Googe and Carron (2016); Karamert et al. (2019). Regarding the formula used for calculation, some studies adopted either of the following formula: “rate = no. of reimplanted CIs/no. of total CIs” or “rate = no. of reimplanted CIs/no. of primary CIs,” in which primary CIs refer to devices inserted into the cochlea for the first time. Thus, the reimplantation rate may be different even for the same batch of patients.

Based on published studies, the CI reimplantation rate ranged from 0.5 to 30% (Beadle et al., 2005; Qiu et al., 2010), and a few studies have reported reimplantation rates higher than 20%. This high reimplantation rate may be attributed to the early publishing time in which the CI surgeries and devices were immature, small sample size, data bias, and immature surgical technology (Hamzavi et al., 2003; Beadle et al., 2005; Kanchanalarp et al., 2005). Patients of different ages with CIs showed different reimplantation rates. The reimplantation rate in children ranged from 0.7 to 30.0% (Kanchanalarp et al., 2005; Sun, 2019), whereas that in adults ranged from 0.4 to 27.3% (Hamzavi et al., 2003; Dağkıran et al., 2020). Some studies have reported that the reimplantation rate in children was significantly higher than that in adults (Sunde et al., 2013; Dağkıran et al., 2020). Children are prone to falls, resulting in head trauma during rapid growth and development (Weise et al., 2005; Wang et al., 2014). The skull and mastoid of children are immature, and rapid growth of the skull can lead to electrode array migration. In addition, the high prevalence of various types of otitis media in children increases the risk of CI failure (Manrique-Huarte et al., 2015). However, some studies have demonstrated no significant differences in the revision rates due to infection complications and device failure rates between adults and children (Sunde et al., 2013; Distinguin et al., 2017). The number of studies that separately calculated the reimplantation rates for children and adults is relatively low; therefore, larger longitudinal cohort studies are required.

### Effect Assessment of Cochlear Reimplantation

The fourth important issue is that the current research mainly focuses on cause analysis and treatment complications. Thus, studies on postoperative effect assessment and the related methods remain limited. Hochmair-Desoyer and Burian (1985) used Bekesy threshold tracking, loudness scaling, open lists of unknown single-syllable words and everyday sentences, and other tests to evaluate the effects of reimplantation in their initial report (Desoyer and Burian, 1985). Since then, almost half of the studies related to reimplantation have mentioned effect evaluations using assessments, such as the Bamford–Kowal–Bench (BKB) test, phonetically balanced kindergarten (PBK) test, Northwestern University number 6 (NU#6) test, common phrases test (CPT), lexical neighborhood test (LNT), categories of auditory performance (CAP), and the speech intelligibility rating (SIR) (Saeed et al., 1995; Beadle et al., 2005; Marlowe et al., 2010; Bhadania et al., 2018). However, these studies have primarily focused on hearing thresholds and speech recognition, and no new assessment methods for neuro-electrophysiological monitoring and evaluation of neurofunctional characteristics have been proposed.

Nevertheless, some new technologies have been demonstrated to be applicable to patients with hearing loss or CIs, such as functional near-infrared optical brain imaging (fNIRS) and electroencephalography (EEG). In Sevy et al. (2010) used NIRS and fMRI to examine the cortical activity in response to auditory stimuli in five children with CIs and five children with normal hearing in Boston Children’s Hospital. In Wang et al. (2020) adopted EEG to evaluate the effect of CIs at three time points after surgery and showed that multiple EEG indices could be used to assess speech perception ability. Many studies have demonstrated that neurological imaging is a safe and feasible approach for the examination of children with CIs, and that it could be an effective method for assessing the effects of reimplanted CIs. Therefore, if these advanced tools could be adopted for the general evaluation of both the first and reimplanted CIs, a better comparison and evaluation of the effect and the regularity of the rehabilitation of cochlear reimplantation can be achieved. In addition, results can be compared between patients with reimplanted CIs and the normal-hearing population, and differences can be used to evaluate the rehabilitation effect.

Regarding the actual effects of reimplantation, Chung et al. (2010) reported that the pure tone audiometry (PTA) and speech recognition scores of all patients who underwent reimplantation for soft failure decreased after reimplantation. Manrique-Huarte et al. (2015) used aided free-field auditory tests. Aided PTA findings after reimplantation improved in 44.4% of patients, deteriorated in 44.4%, and showed no significant difference in 11.1%, whereas speech recognition scores improved in 63.6%, showed no significant change in 9.1%, and worsened in 27.3%. In a study on the performance of 56 children 18 months after cochlear reimplantation, 87% showed better speech perception, 10% reported similar results, and 3% showed worse speech perception after surgery (Marlowe et al., 2010). Younger children were more likely to achieve or exceed their previous best performance than older children. The age at reimplantation, interval between the initial implantation and reimplantation, auditory input during the interval, depth of the electrode array, device activation, and device upgrade also influenced the postoperative effects (Marlowe et al., 2010; Lenarz, 2017; Roßberg et al., 2021). However, no single study has systematically discussed these factors. In addition, only a few effect evaluations have been carried out, with effect evaluation analysis not performed in some large-scale studies. Therefore, future studies should utilize more advanced assessment tools, such as functional near-infrared spectroscopy and electroencephalography, and should take more factors into consideration to systematically assess the rehabilitation effect and explore the internal patterns.

### Study Scale and the Influence of the Reimplantation Side

Most studies on cochlear reimplantation were single-center studies with a relatively small sample size, and multicenter studies only accounted for 6% (Chung et al., 2010; Hermann et al., 2020). Therefore, the use of rigorous and advanced statistical analysis methods to obtain more robust findings is difficult. Moreover, in the absence of a standard criterion for the selection of the reimplantation side, most studies selected the side based on infection, cochlear ossification, and deformity (Chung et al., 2010; Lu and Cao, 2014; Manrique-Huarte et al., 2015), without considering other influencing factors, such as the duration between the first implantation and reimplantation and continuous auditory input during the period. Thus, integration of the patient resources of several hospitals and multicenter studies should be performed to elevate the level of evidence on this topic.

## Conclusion

As the use of CIs continues to increase worldwide and the service life of early implanted devices approaches its end, the number of reimplanted CIs has increased in recent years. However, CI reimplantation is associated with some limitations, such as non-standard definitions and calculation formula for the reimplantation rate and the absence of high-quality studies on rehabilitation effect. Thus, establishment of a standard definition and appropriate scope for future studies is important. Longitudinal and multicenter studies should be conducted using more advanced tools and after adjusting for more covariates to systematically assess the effects of reimplantation and develop an effective system for follow-up and evaluation.

## Author Contributions

XY and HL made substantial contributions to the study conception and design, literature research, and drafting and revision of the manuscript. JS provided critical insights and points regarding the conception of cochlear reimplantation and performed draft revision. XD provided substantial suggestions for the definition and status of cochlear reimplantation and critically revised the manuscript for important intellectual content. YZa made substantial contributions to this study, including raising on-the-mark suggestions to the definition and status of cochlear reimplantation, sharing the surgery details of cochlear implantation and reimplantation, and revising the manuscript critically for important intellectual content. YZe made substantial contributions to the conception and design of this study, especially in the effect assessment of cochlear reimplantation, and was involved in critically revising the manuscript for important intellectual content.

## Conflict of Interest

The authors declare that the research was conducted in the absence of any commercial or financial relationships that could be construed as a potential conflict of interest.

## Publisher’s Note

All claims expressed in this article are solely those of the authors and do not necessarily represent those of their affiliated organizations, or those of the publisher, the editors and the reviewers. Any product that may be evaluated in this article, or claim that may be made by its manufacturer, is not guaranteed or endorsed by the publisher.
